# Aberrant Expression of PHLPP1 and PHLPP2 Correlates with Poor Prognosis in Patients with Hypopharyngeal Squamous Cell Carcinoma

**DOI:** 10.1371/journal.pone.0119405

**Published:** 2015-03-20

**Authors:** Jieyu Zhou, Xuemin Yu, Juan Wang, Tao Li, Tong Jin, Dapeng Lei, Xinliang Pan

**Affiliations:** Department of Otorhinolaryngology, Qilu Hospital of Shandong University, Jinan, Shandong Province, P. R. China; Ohio State University Medical Center, UNITED STATES

## Abstract

The PHLPP (pleckstrin homology [PH] domain leucine rich repeat protein phosphatase) family, which represents a family of novel Ser/Thr protein phosphatases, is composed of 2 members: PHLPP1 and PHLPP2. PHLPPs partake in diverse cellular activities to exhibit their antitumor and metastasis suppressor functions. It is necessary to investigate the expression patterns of PHLPP1 and PHLPP2 in hypopharyngeal squamous cell carcinomas (HSCCs) and clarify their clinical significance. A total of 138 patients with primary HSCC who underwent curative surgical treatment as an initial treatment were enrolled in this study. A total of 138 HSCC specimens and 64 adjacent noncancerous mucosal epithelial tissues were collected. The expression levels of PHLPP1 and PHLPP2 were examined by quantitative reverse transcription polymerase chain reaction and immunohistochemistry assays. Correlations between clinicopathological parameters of the patients were further evaluated. PHLPP1 and PHLPP2 mRNA transcript levels were significantly lower in tumor samples than in paired adjacent nontumor mucosae (*P*<0.0001, both). Positive correlations were observed between the mRNA levels of PHLPP1 and PHLPP2 in HSCC tissues (correlation coefficient *r* = 0.678, *P*<0.001) and in adjacent nontumor mucosae (*r* = 0.460, *P*<0.001). The majority of the noncancerous tissues showed high expression levels of PHLPP1 (87.5%, 56/64) and PHLPP2 (85.9%, 55/64). However, the expressions of PHLPP1 and PHLPP2 were significantly decreased in 83.3% (115/138) and 82.6% (114/138) of tumor tissues, respectively (*P*<0.0001, both). The expressions of both PHLPP isoforms were significantly related to the tumor clinical stage, differentiation, and cervical lymph node metastasis (*P*<0.05, all). It was PHLPP1 but not PHLPP2 that was significantly related to the tumor T stage. Low PHLPP1 and PHLPP2 expressions were associated with poor overall survival (OS) in HSCC patients (*P* = 0.004, *P* = 0.008, respectively). Multivariate analysis revealed that PHLPP1 was an independent prognostic factor for OS. This study indicates that, in HSCC, aberrant expressions of PHLPP1 and PHLPP2 are common events, and loss of PHLPPs might identify patients with poor prognostic outcomes.

## Introduction

Hypopharyngeal squamous cell carcinomas (HSCCs) account for approximately 5–15% [1] of all head and neck cancers; they are the most aggressive and have the worst prognosis in the head and neck area [2]. At present, the main treatment strategy for HSCC continues to be surgery followed by radiotherapy. Despite the improvements made in recent years, no treatment achieves a satisfactory therapeutic outcome for patients, and the 5-year survival rate is estimated to be at 25–40% [2]. The poor prognosis of HSCC might be because of the lack of early detection and high rate of metastasis [3]. Many molecules, such as SIRT1, DBC1 [4], Beclin-1, LC3 [5], and Caveolin-1 [6], have been evaluated as candidate biomarkers for HSCC, but none have been widely used in practice because of the lack of understanding of the molecular mechanisms involved in HSCC development, progression, and treatment response [7]. Therefore, studies of novel and more effective molecular biomarkers of HSCC prognosis and progression are necessary.

The PHLPP (pleckstrin homology [PH] domain leucine-rich repeat [LRR] protein phosphatase) family, which represents a family of novel Ser/Thr protein phosphatases, is composed of 2 members: PHLPP1 and PHLPP2 [8,9]. Mapped to chromosome 18q21.33 and 16q22.3–16q23.1 respectively, PHLPP1 and PHLPP2 are almost identical in domain structure. Both contain an *N*-terminus Ras association domain, followed by a PH domain, LRR, PP2C domain, and PDZ binding motif [10]. PHLPPs partake in diverse cellular activities, such as survival, proliferation, migration, quiescence, and apoptosis, to exert their antitumor and metastasis suppressor functions [8,9,11]. The expressions of PHLPPs are frequently decreased in a variety of human cancers, such as prostate cancer [12], colon cancer [13], breast cancer [14], melanoma [15], ovarian cancer [16], and Wilms tumor [17]. However, the expression and functional significance of PHLPPs in HSCC progression have not been characterized.

The aim of this study was to test whether the expression levels of PHLPPs could be potential biomarkers for HSCC. To address this issue, the expression patterns of PHLPPs in HSCC patients were first examined and their clinicopathological features were assessed. Secondly, survival analysis was used to investigate their prognostic values.

## Materials and Methods

### Ethics Statement

The study protocol was approved by Ethics Boards of Qilu Hospital (protocol no. 1180), and tissue specimen acquisition was carried out in accordance with the institutional guidelines. All patients signed written informed consent, and the study followed the Declaration of Helsinki.

### Patients and tissue specimens

A total of 138 patients with primary HSCC who underwent curative hypopharyngectomy as an initial treatment at the Department of Otorhinolaryngology of Qilu Hospital of Shandong University, Jinan, China, from August 2009 to December 2013 were enrolled in this study. Clinical data were obtained at initial presentation and through follow-up. At initial presentation, all patients completed an epidemiological questionnaire including data on alcohol and smoking status. Drinking status was categorized as “ever-drinkers” (those who had drunk at least one alcoholic beverage per day for at least 1 year during their lifetime) and “never-drinkers” (those who never had such a pattern of drinking) [18]. Smoking status was categorized as “ever-smokers” (those who had smoked at least 100 cigarettes in their lifetime) and “never-smokers” (those who had smoked <100 cigarettes in their lifetime) [18]. All patients underwent neck dissection, no matter whether the cervical lymph nodes metastasis diagnosis before surgery exited or not. All patients received adjuvant radiotherapy (50–75 Gy) for 20 days to 2 months after surgery. Patients who had received neoadjuvant chemotherapy or radiation therapy before surgery were excluded from this study.

A total of 138 malignant tumors and 64 matched non-cancerous mucosal epithelial tissues were collected during curative resection. Fresh specimens were divided into two parts, one part was stored in liquid nitrogen immediately for total RNA extraction, the other was fixed in 10% buffered formaldehyde for histological evaluation. Diagnosis and histological typing were carried out by two experienced pathologists according to the Word Health Organization classification. Tumor staging was in accordance with the International Union against Cancer (UICC, 2002) TNM classification.

### RNA Extraction and Quantitative Real-time PCR (qRT-PCR)

Total RNA was extracted using Trizol reagent (Takara, Dalian, China) and then was reverse-transcribed to cDNA using PrimeScript Reverse Transcriptase (Takara, Dalian, China) following the manufacturer’s protocol. RNA concentration was quantified by NanoDrop ND-1000 (NanoDrop Technologies/Thermo Scientific, Wilmington, DE, USA), and RNA integrity was assessed by standard denaturing agarose gel electrophoresis. qRT-PCR was performed using the SYBR Green chemistry in the ABI 7900HT Sequence Detection (ABI Applied Biosystems, Foster City, CA). The gene-specific primers used were as follows: PHLPP1, 5’-AGGCGCATGCACACCGTGAT-3’ (sense), 5’-GGACAAGGCGCGGGTTTCCA-3’ (antisense); PHLPP2, 5’-ATGGAGCAGACACTACCACTG-3’ (sense), 5’-GCAAAGGACGAGATGTAAGTCA-3’ (antisense); GAPDH, 5’-GGAGCGAGATCCCTCCAAAAT-3’ (sense), 5’-GGCTGTTGTCATACTTCTCATGG-3’ (antisense). qRT-PCR was performed in a 10-uL reaction volume and consisted of an initial denaturation step at 95°C for 30 sec followed by amplification with 40 cycles at 95°C for 5 sec and 60°C for 30 sec. The threshold cycle (Ct) was defined as the cycle number at which the fluorescence passed a pre-determined threshold. Both target and reference (GAPDH) genes were amplified in separate wells in triplicate. Gene expression was calculated using the comparative threshold cycle (2^−ΔΔCT^) method.

### Immunohistochemistry (IHC) Staining

**Fig 1:**
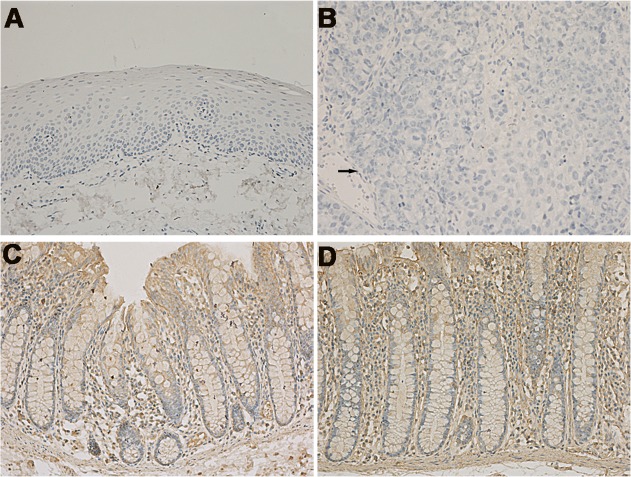
Negative and positive controls of PHLPP1 and PHLPP2 expression under microscope (×200). Negative control of (A) noncancerous hypopharyngeal mucosa and (B) HSCC. Positive control of (C) PHLPP1 and (D) PHLPP2 expression in normal colonic mucosa. Arrows designate regions of tumor.

Fresh specimens were embedded in paraffin and sectioned at 4μm with a Leica microtome (Leica, Wetzlar, Germany). The immunohistochemistry procedure was performed according to the manufacturer’s instructions. The sections were deparaffinized and rehydrated. Then the sections were treated with a microwave antigen retrieval procedure in citrate buffer (PH 6.0) before being immersed in 3% H_2_O_2_ to block endogenous peroxidase. Non-specific binding was blocked with 10% goat serum for 15 min. Sections stained with primary antibodies were incubated at 4°C overnight. The following primary antibodies were used: anti-PHLPP1 (1:500 dilution; ab71972, Abcam, Cambridge, UK), anti-PHLPP2 (1:50 dilution; ab77665, Abcam, Cambridge, UK). Slides were then incubated with a biotinylated secondary antibody (Zsbio, Beijing, China) at 37°C for 15 min, and then with peroxidase-conjugated streptavidin for 15 min. DAB (3, 3-diaminobenzidine) (Zsbio, Beijing, China) was used as a substrate for color development. Slides were counterstained with Mayer’s hematoxylin. For negative control, PBS buffer was used to replace the primary antibodies (Fig. 1A, B). For positive control, normal colonic mucosa slide was used [13] (Fig. 1C, D).

Sections were photographed under light microscope (Leica DM2500, Germany), and images were captured with a digital camera (Leica DFC295, Germany). The immunohistochemical analysis was performed by two pathologists in a double-blind analysis. The result of staining was scored according to the intensity of staining and the percentage of stained cells. The semi-quantification for immunostaining intensity was scored according to the following scale: 0, no staining; 1, mild staining; 2, moderate staining; and 3, marked staining. Average numbers of immunopositive cells within the samples were determined in at least five areas at ×200 magnification. The percentage of immunopositive cells was scored on a scale of 0 (0–9%), 1 (10–25%), 2 (26–50%), 3 (51–75%) and 4 (≥76%) [4,13,19]. For each sample, the scores from the two scoring systems were multiplied to get a final point score. The maximum combined score was 12 and the minimum combined score was 0. So the immunohistochemical expression of PHLPP1 and PHLPP2 was defined according to the following scores; negative, combined score 0; low expression, combined score 1–8; high expression, combined score 9–12.

### Follow-up of patients

The time of follow-up was calculated from the date of operation. Follow-up data were obtained by phone, letter, and the outpatient clinical database. All patients were subjected to close follow-up observation every 3 months for the first year, and every 6 months next. The median patient follow-up duration after resection was 31.4 months (range, 7–53 months). Demographic and clinical data were collected respectively.

### Statistical Analysis

SPSS 17.0 (SPSS Inc., Chicago, IL, USA) and GraphPad Prism 5.0 (GraphPad Software Inc., San Diego, CA, USA) statistical software were employed for the analysis. Wilcoxon matched pairs test was used to compare the mRNA expression levels in tumor vs. nontumor tissues. Mann-Whitney 2-tailed test was used to compare the protein expression levels in tumor vs. nontumor tissues. The Spearman rank analysis was used to analyze the correlation between PHLPP1 and PHLPP2 levels. The association between categorical variables was analyzed by the Pearson Chi-square tests or continuous correction Chi-square tests as appropriate. Overall survival curves were drawn using Kaplan-Meier method, and the differences between the survival curves were examined using the log-rank test. The Cox univariate and multivariate analyses were performed to explore the influences of different prognostic factors on overall survival (OS) time. OS was described as the time from surgery to the date of the patient’s death or the date of last follow-up. Data are presented as mean ± standard deviation (SD). In all analyses, a two-sided *P* value <0.05 was considered statistically significant.

## Results

### Demographic and Clinicopathological Characteristics

Patient clinicopathological information and demographic data are shown in Table 1. In summary, the study cohort mainly consisted of male patients (91.3%), with a median age of 59.5 years (range: 38–79 years), with predominantly more advanced T-stage (T3-T4 in 53.6%), lymph node metastasis (78.3%), and advanced clinical stage (83.3% stage III and IV). No patients presented distant metastases. Pathological studies confirmed well differentiation in 26 cases (18.9%), moderate differentiation in 62 cases (44.9%), and poor differentiation in 50 cases (36.2%).

**Table 1:** Patient clinicopathological information and demographic data (n = 138).

Variable	No.(%)
**Age at presentation**	
Median	59.5
Range	38–79
**Gender**	
Female	12 (8.7)
Male	126 (91.3)
**Primary location**	
Sinus piriformis	99 (71.7)
Posterior pharyngeal wall	27 (19.6)
Postcricoid area	12 (8.7)
**T category**	
T1-T2	64 (46.4)
T3-T4	74 (53.6)
**Node metastasis**	
N0	30 (21.7)
N1	51 (37.0)
N2	49 (35.5)
N3	8 (5.8)
**M category**	
M0	138 (100.0)
M1	0 (0.0)
**Clinical stage**	
I-II	23 (16.7)
III-IV	115 (83.3)
**Histologic differentitation**	
Well	26 (18.9)
Moderate	62 (44.9)
Poor	50 (36.2)
**Treatment**	
surgery plus radiotherapy	138 (100.0)
**Death**	
Yes	78 (56.5)
No	60 (43.5)

### qRT-PCR Analysis for the Expression of Both PHLPP Isoforms in Carcinomas and Noncancerous Mucosae

**Fig 2:**
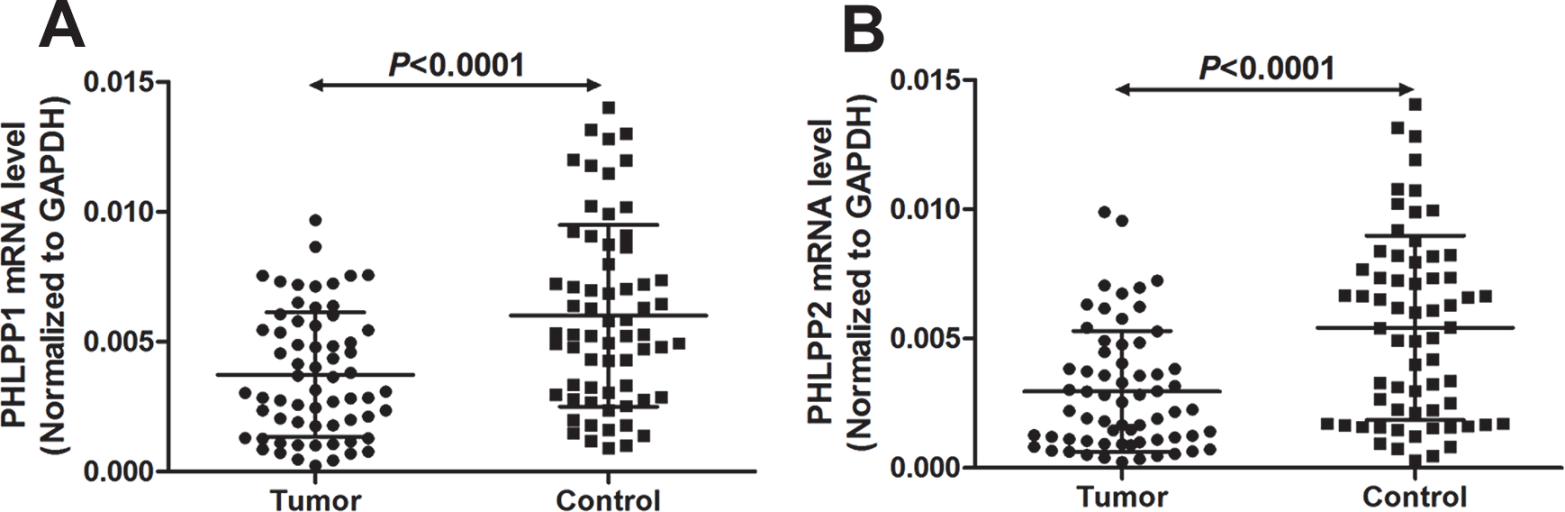
Quantitative determination of mRNA by qRT-PCR for (A) PHLPP1 and (B) PHLPP2 in noncancerous mucosae and carcinomas. Target RNA relative expression levels were given as ratios of GAPDH transcript levels in the same RNA sample. Scatter plots were shown with mean ± standard deviation (SD). Statistical analyses were performed with the Wilcoxon matched paired test (2-tailed).

**Fig 3:**
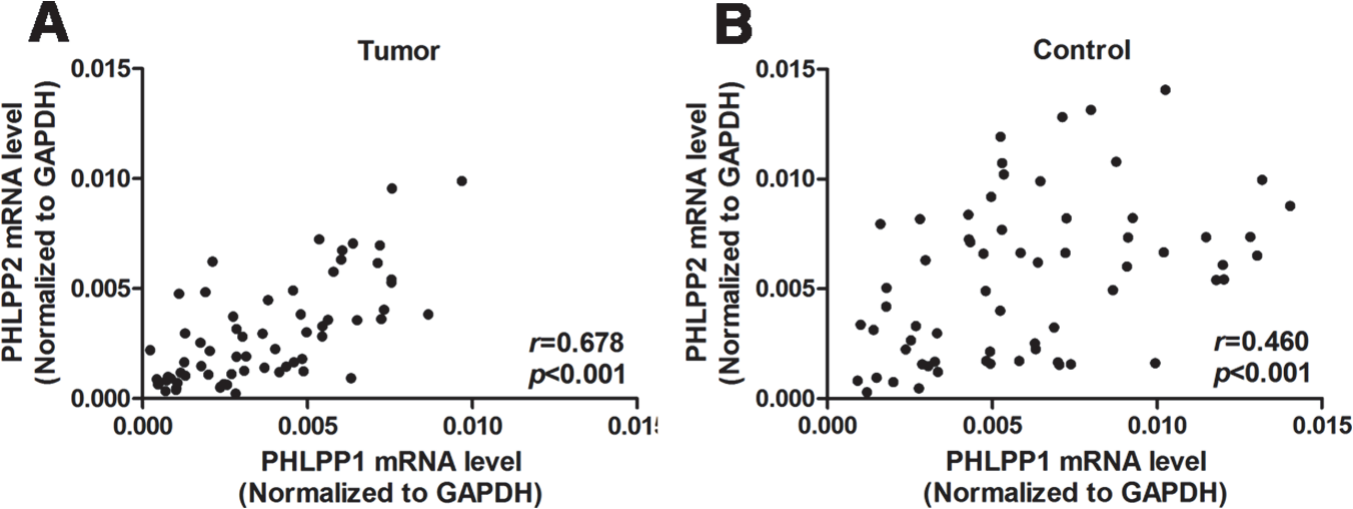
Relationship between mRNA levels of PHLPP1 versus PHLPP2 in (A) tumors and (B) adjacent non-cancerous mucosae. The correlation coefficient (*r*) was calculated by using Spearman rank analysis.

The mRNA expression levels of PHLPP1 and PHLPP2 in 64 paired HSCC specimens and noncancerous mucosal epithelial tissues were quantitatively determined. The relative expression levels of target mRNA were presented as ratios of GAPDH transcript levels in the same RNA sample. Our quantitative reverse transcription polymerase chain reaction (qRT-PCR) assays revealed that the mean mRNA levels of PHLPP1 were 0.0037 ± 0.0024 in HSCC tissues and 0.0060 ± 0.0035 in adjacent nontumor mucosae (*P*<0.0001, Fig. 2A). The mean PHLPP2 mRNA levels were 0.0030 ± 0.0023 in HSCC tissues and 0.0054 ± 0.0036 in adjacent nontumor mucosae (*P*<0.0001, Fig. 2B). So, the expressions of both PHLPP1 and PHLPP2 mRNAs in tumor samples were much lower than in adjacent nontumor mucosae. Moreover, positive correlations were observed between the mRNA levels of PHLPP1 and PHLPP2 in HSCC tissues (correlation coefficient *r* = 0.678, *P*<0.001, Fig. 3A) and in adjacent nontumor mucosae (*r* = 0.460, *P*<0.001, Fig. 3B).

### Immunohistochemical Analysis for the Expression of PHLPP1 and PHLPP2 in HSCC tissues and Adjacent Nontumor Tissues

**Fig 4:**
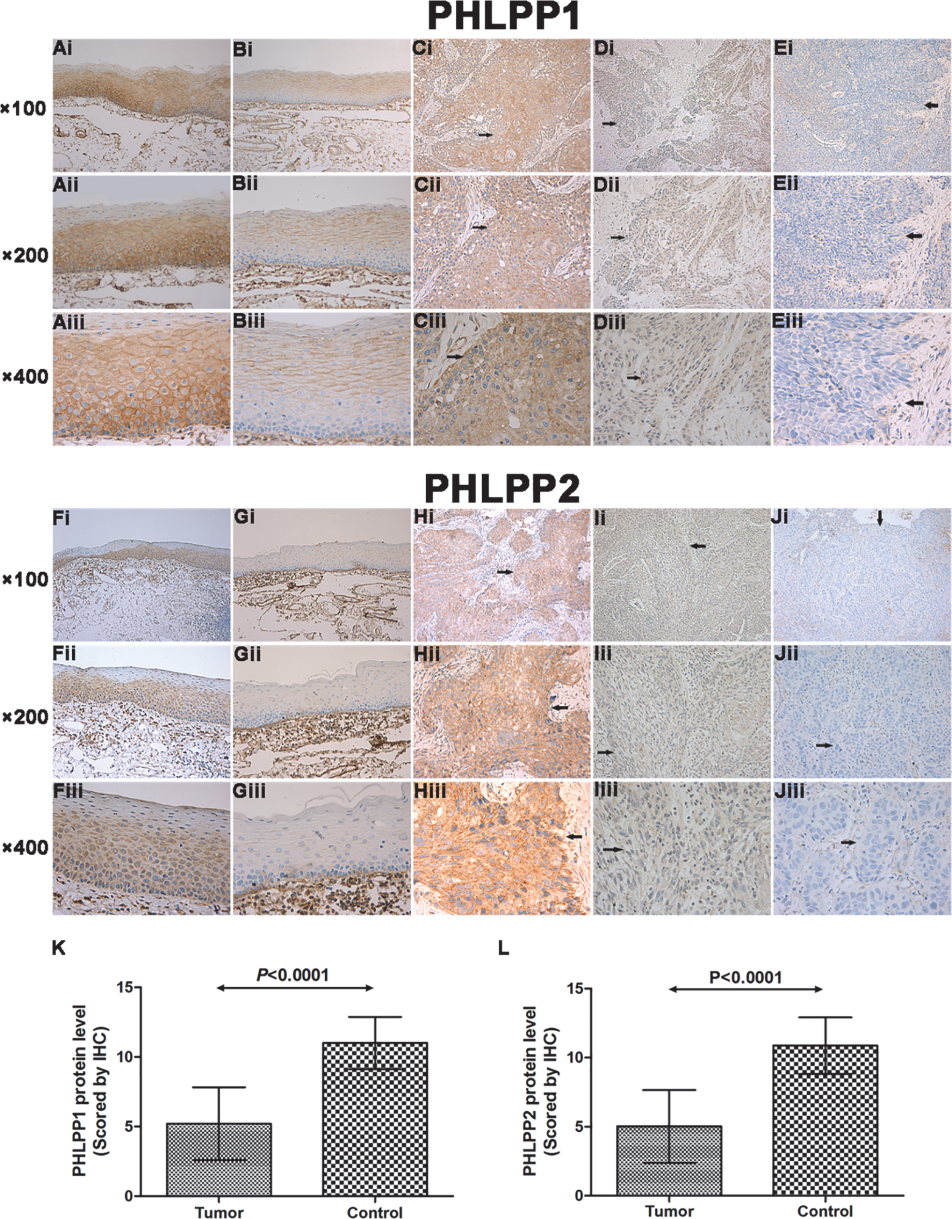
Immunohistochemical staining of PHLPP1 and PHLPP2 expression in non-cancerous mucosae and carcinomas under microscope (×100, ×200, ×400). High PHLPP1 expression in (Ai–iii) noncancerous mucosae and (Ci–iii) carcinomas. High PHLPP2 expression in (Fi–iii) noncancerous mucosae and (Hi–iii) carcinomas. (Di–iii) Low PHLPP1 and (Ii–iii) PHLPP2 expression in carcinomas. Negative PHLPP1 expression in (Bi–iii) noncancerous mucosae and (Ei–iii) carcinomas. Negative PHLPP2 expression in (Gi–iii) noncancerous mucosae and (Ji–iii) carcinomas. Expression of (K) PHLPP1 and (L) PHLPP2 protein was determined by immunohistochemistry. Results were shown as mean ± SD. Statistical analyses were performed with Mann–Whitney 2–tailed test. Arrows designate regions of tumor.

Using immunohistochemistry staining, it was found that both PHLPP isform proteins were observed mainly in the membrane and cytoplasm of HSCC cells and adjacent noncancerous squamous epithelial cells. The majority of the noncancerous tissues showed high expression levels of PHLPP1 (87.5%, 56/64) and PHLPP2 (85.9%, 55/64). In marked contrast, the expressions of PHLPP1 and PHLPP2 were significantly decreased in 83.3% (115/138) and 82.6% (114/138) of tumor tissues, respectively (*P*<0.0001, Fig. 4K, L). The results revealed that the expression of either PHLPP1 or PHLPP2 was significantly decreased in carcinomas relative to adjacent noncancerous epithelial tissues. In addition, the intensity staining score between the 2 isoforms was positively correlated with each other in carcinomas (*r* = 0.873, *P*<0.001, Table 2), suggesting that loss of both PHLPP isoforms was commonly observed in the same patient.

**Table 2:** Spearman correlation between PHLPP1 and PHLPP2 using staining score.

Group	No.	Spearman correlation (*r*)	*P* value
**HSCC tissues**	138	0.873	<0.001
**Non-cancerous tissues**	64	0.850	<0.001

*P* values were determined by Spearman rank analysis. HSCC, hypopharyngeal squamous cell carcinoma.

### Association of PHLPP1 and PHLPP2 Protein Expression with Clinicopathological Features

As shown in Table 3, the expression levels of both PHLPP isoforms were significantly related to the tumor clinical stage, differentiation, and cervical lymph node metastasis (all *P*<0.05). It was PHLPP1 but not PHLPP2 that was significantly related to the tumor T stage (*P* = 0.047). Other variables, such as age, gender, tobacco exposure, and alcohol consumption, did not showed any statistically significant association with the expression levels of PHLPP1 and PHLPP2.

**Table 3:** Clinicopathologic variables and the protein expression status of PHLPP1 and PHLPP2.

Characteristics	No.	PHLPP1 expression n (%)		*P* value	PHLPP2 expression n (%)		*P* value
		Low	High		Low	High	
**Gender**				1.000			0.640
Male	126	105 (83.3)	21 (16.7)		103 (81.7)	23 (18.3)	
Female	12	10 (83.3)	2 (16.7)		11 (91.7)	1 (8.3)	
**Age (years)**				0.819			0.653
<60	69	57 (82.6)	12 (17.4)		58 (84.1)	11 (15.9)	
≥60	69	58 (84.1)	11 (15.9)		56 (81.2)	13 (18.8)	
**Tobacco**				0.540			0.637
Never	35	28 (80.0)	7 (20.0)		28 (80.0)	7 (20.0)	
Ever	103	87 (84.5)	16 (15.5)		86 (83.5)	17 (16.5)	
**Alcohol**				1.000			0.906
Never	30	25 (83.3)	5 (16.7)		25 (83.3)	5 (16.7)	
Ever	108	90 (83.3)	18 (16.7)		89 (82.4)	19 (17.6)	
**Differentiation**				0.001*			0.006*
Well	26	16 (61.5)	10 (38.5)		17 (65.4)	9 (34.6)	
Moderate	62	51 (82.3)	11 (17.7)		50 (80.6)	12 (19.4)	
Poor	50	48 (96.0)	2 (4.0)		47 (94.0)	3 (6.0)	
**T stage**				0.047*			0.081
T1-T2	64	49 (76.6)	15 (23.4)		49 (76.6)	15 (23.4)	
T3-T4	74	66 (89.2)	8 (10.8)		65 (87.8)	9 (12.2)	
**Clinical stage**				0.001*			0.035*
I-II	23	13 (56.5)	10 (43.5)		15 (65.2)	8 (34.8)	
III-IV	115	102 (88.7)	13 (11.3)		99 (86.1)	16 (13.9)	
**LN metastasis**				0.002*			0.007*
N0	30	18 (60.0)	12 (40.0)		21 (70.0)	9 (30.0)	
N1	51	46 (90.2)	5 (9.8)		40 (78.4)	11 (21.6)	
N2	49	45 (91.8)	4 (8.2)		47 (95.9)	2 (4.1)	
N3	8	6 (75.0)	2 (25.0)		6 (75.0)	2 (25.0)	

*P* values were determined by Pearson Chi-square tests or continuous correction Chi-square tests. LN, lymph node.

*P<0.05

### Survival Analysis and Prognostic Significance of PHLPP1 and PHLPP2 Protein Expression

**Fig 5:**
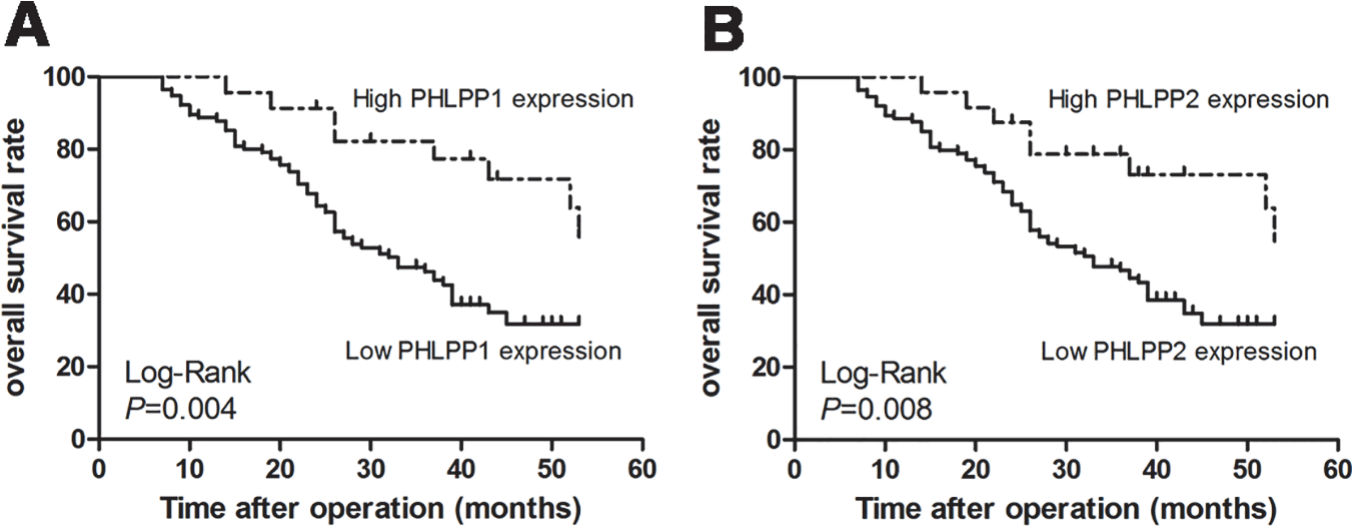
Kaplan-Meier curves for PHLPP1 and PHLPP2 expression in 138 patients with HSCC. Low level of (A) PHLPP1 (*P* = 0.004) and (B) PHLPP2 (*P* = 0.008) was associated with poor prognosis.

The prognostic values of PHLPP1 and PHLPP2 protein expression in HSCC patients was then determined. The Kaplan-Meier analysis showed that the overall survival (OS) rates of patients with low PHLPPs was significantly lower than that of patients with high PHLPPs (*P* = 0.004, *P* = 0.008, respectively, Fig. 5A, B). The 3-year OS rates for patients with low and high levels of PHLPP1 were 46.2% and 82.2%, respectively. The 3-year OS rates for patients with low and high levels of PHLPP2 were 46.7% and 78.8%, respectively. On univariate Cox regression analysis, cervical lymph node metastasis, T stage, differentiation, clinical stage, and PHLPP1 and PHLPP2 protein levels were all confirmed as prognostic factors for OS, whereas other clinical indexes, such as sex, age, tobacco exposure, and alcohol consumption had no prognostic significance for OS (Table 4). In addition, in the multivariate Cox regression analysis, cervical lymph node metastasis (*P* = 0.042, hazard ratio [HR] 1.617, 95% confidence interval [CI]: 1.018–2.567; Table 4), T stage (*P* = 0.035, HR 1.665, 95% CI: 1.035–2.679; Table 4) and PHLPP1 protein expression (*P* = 0.018, HR 0.402, 95% CI: 0.189–0.854; Table 4) were independent prognostic factors. However, differentiation, clinical stage, and PHLPP2 protein level were not determined as independent prognostic indicators.

**Table 4:** Results of univariate and multivariate Cox regression analyses in 138 patients.

Parameter	Unfavorable vs. favorable	Univariate analysis			Multivariate analysis		
		HR	95% CI	*P* value	HR	95% CI	*P* value
**Age (years)**	<60 vs. ≥60	1.003	0.643–1.565	0.989			
**Gender**	Female vs. male	1.293	0.622–2.688	0.492			
**Smoking**	Never vs. Ever	1.152	0.680–1.952	0.599			
**Alcohol**	Never vs. Ever	1.153	0.656–2.025	0.621			
**Node metastasis**	N0-N1 vs. N2-N3	1.914	1.219–3.006	0.005*	1.617	1.018–2.567	0.042*
**T stage**	T1-T2 vs. T3-T4	1.979	1.243–3.150	0.004*	1.665	1.035–2.679	0.035*
**Differentiation**	Poor vs. well and moderate	1.688	1.073–2.656	0.023*	1.532	0.963–2.436	0.072
**Clinical stage**	I-II vs. III-IV	1.949	1.013–3.748	0.046*	0.784	0.340–1.805	0.567
**PHLPP1 expression**	Low vs. high	0.353	0.167–0.746	0.006*	0.402	0.189–0.854	0.018*
**PHLPP2 expression**	Low vs. high	0.385	0.184–0.808	0.012*	0.737	0.312–1.742	0.487

HR, hazard ratio; CI, confidence interval.

*P<0.05

## Discussion

PHLPP, a novel member of type 2C family of protein phosphatases [20], is now known to potently suppress cell survival by both inhibiting proliferative pathways and by promoting apoptotic pathways. In the former, PHLPPs directly dephosphorylate the hydrophobic motif on Akt [8,9], protein kinase C (PKC) [21], and ribosomal protein S6 kinase (S6K) [22], thereby terminating the signaling by these prosurvival kinases. In the latter, PHLPPs dephosphorylate and thus activate the proapoptotic kinase mammalian sterile 20-like kinase 1 (Mst1) [23], thereby promoting apoptosis. Furthermore, PHLPPs decrease protein synthesis and cell size by a mechanism dependent on the downregulation of S6K [20,22]. As novel tumor suppressor genes, PHLPPs present with loss of or reduced expression in many cancers [12–17]. However, it has been reported that PHLPPs-dependent inhibition of cell growth and apoptosis may be cell- and cancer-type specific [8,9,13,23]. Therefore, it is necessary to investigate whether PHLPPs are involved in the development of HSCC.

In this study, the expression patterns of both PHLPP isoforms in HSCC specimens and adjacent noncancerous mucosae at both mRNA and protein levels were examined. The expression levels of PHLPP1 and PHLPP2 were found to be lost or decreased in carcinomas compared with the adjacent noncancerous mucosae, which was certificated by qRT-PCR and immunohistochemical staining. The results also demonstrated that both PHLPP protein isoforms were decreased commonly in the same patient, which is consistent with previously findings on colon cancer by Jianyu Liu et al. [13]. Therefore, the 2 PHLPP isoforms might function together to inhibit tumorigenesis. In addition, the decreased expression of both PHLPP proteins in carcinomas was correlated with poor differentiation, clinical stage, and cervical lymph node metastasis. Moreover, decreased PHLPP1 protein expression was significantly related to the advanced T stage. Therefore, the decreased expressions of PHLPP1 and PHLPP2 might promote the invasive behaviors as well as metastasis of HSCC. Furthermore, survival analysis revealed that the expression level of PHLPPs was significantly associated with patient survival: patients with low PHLPP1 and PHLPP2 protein expressions have a poor prognosis, and PHLPP1 was an independent prognostic factor. Therefore, these results might help us to determine the prognosis of patients according to the expression levels of PHLPP1 and PHLPP2.

Metastasis is the main reason for the poor prognosis of HSCC [2]. Increasing evidence supports the contribution of phosphoinositide 3-kinase (PI3K)/Akt signaling pathway to cancer progression [24–29]. Signaling through this pathway is often increased in primary and metastatic tumors [25,30]. Many molecular players in this pathway are reported to act as either tumor suppressors or oncogenes. Although much attention has been focused on the factors that positively affect this pathway, negative regulation is equally important [8,31–33]. Several inhibitors of the pathway targeted at PI3K/Akt are currently in clinical trials as cancer therapeutics [25]. PTEN tumor suppressor, which directly antagonizes the activity of PI3K by dephosphorylating PIP3, is an upstream inhibitor of this pathway [34]. PHLPPs, another PI3K/Akt signaling pathway inhibitors, exerted their tumor-suppressing functions by dephosphorylation and inactivation of Akt on Ser^473^ [35]. Liao et al. reported that microRNA-224 promotes cell proliferation and tumor growth in human colorectal cancer by repressing PHLPP1 and PHLPP2 [36]. Wang et al. reported that loss of PHLPP1 expression correlates with lymph node metastasis and exhibits a poor prognosis in patients with gastric cancer [37]. Rakha proposed that decreased PHLPP1 expression correlates with increased metastatic potential in breast cancer cells [17]. In our previous study, we have indicated that PTEN is decreased in many HSCC patients, and it might play an important role in the progression of HSCC [38]. Here, we noticed a significant correlation of decreased PHLPPs with the invasion of regional lymph nodes, advanced clinical stage, and poor prognosis in HSCC patients. Therefore, PHLPPs might be important biomarkers and novel therapeutic molecules. To the best of our knowledge, this is the first clinical report to investigate the prognostic value of the expressions of PHLPPs in HSCC patients. However, this retrospective study was confined to HSCC patients who underwent curative resection at our center. In the future, studies of larger patient scale, and mechanisms of low expression of PHLPPs in HSCC will be explored.

## Conclusion

Herein, we reported novel dysregulated Ser/Thr protein phosphatases in HSCC: the expressions of PHLPPs were decreased in HSCCs than in adjacent noncancerous tissues. The expressions of PHLPPs significantly correlated with tumor differentiation, clinical stage, and cervical lymph node metastasis. Furthermore, we also demonstrated that low expressions of PHLPPs were predictors for survival in patients with HSCC, and PHLPP1 was an independent prognostic factor. These results suggested that PHLPPs could be novel therapeutic molecules for treatment of HSCC patients in the future.
